# Revealing the molecular interplay of curcumin as *Culex pipiens* Acetylcholine esterase 1 (AChE1) inhibitor

**DOI:** 10.1038/s41598-021-96963-8

**Published:** 2021-09-01

**Authors:** Priyashi Rao, Dweipayan Goswami, Rakesh M. Rawal

**Affiliations:** 1 Department of Biochemistry & Forensic Science, University School of Sciences, Gujarat University, Ahmedabad, Gujarat 380009 India; 2 Department of Microbiology & Biotechnology, University School of Sciences, Gujarat University, Ahmedabad, Gujarat 380009 India

**Keywords:** Malaria, High-throughput screening, Protein structure predictions, Enzyme mechanisms

## Abstract

Emergence of vector borne diseases has continued to take toll on millions of lives since its inception. The use of insecticides began as vector control strategy in the early 1900’s but the menace of insects is still prevalent. Additionally, the inadequate use of organophosphates and carbamates which target acetylcholine esterase (AChE), are known to develop resistance amongst vectors of transmission and are toxic to humans. In this study, extensive computational screening was performed using homology modelling, molecular docking, molecular dynamics (MD) simulation and free energy change calculation, which highlighted curcumin as a lead molecule out of ~ 1700 phytochemicals against *Culex pipiens* AChE. In vivo larvicidal activity was carried out along with in vivo and in vitro AChE inhibition assay to determine the biochemical efficacy of curcumin. Our study reveals that curcumin induces mortality in *Cx. pipiens* at an early stage of its life cycle by AChE inhibition. This also underlines the use of curcumin as a coming-age natural product insecticide.

## Introduction

Vector borne disease are one of the major health problems in the world, accounting for more than 17% of all infectious diseases (World Health Organisation, 2018)^1^. Mosquitoes are the vectors that spread infectious diseases to various life forms including humans. Many of these mosquito vectors are bloodsucking macroscopic flying creatures that ingest disease-causing pathogens during a blood meal from an infected host (human or animal) and then spread them to a new host, resulting in a chain of severe life-threatening infections^2^. When a vector (like mosquito) gets infected, it is also capable of spreading the pathogen for the entirety of its life onto each subsequent bite/blood meal. *Culex pipiens* are known to feed on variety of hosts thereby amplifying the pathogenic cross-infectivity, causing major vector-borne disease like avian malaria, filariasis, Japanese encephalitis and west nile virus infection amongst many others^3^. Starting since early 1900’s, manufacturing of chemical insecticides became a crucial aspect of insect pest management^4^. Insecticides are known to inhibit the functionality of a specific biochemical bioprocess or protein of the insect system at different stages of mosquitoe’s life cycle; Acetylcholine esterase (AChE) is one such protein of importance involved in neurotransmission, therefore considered as a well-known target for insecticides belonging to the class of organophosphates and carbamates.

Generally, at the presynaptic neuron, the enzyme acetyltransferase catalyses the formation of Acetylcholine (ACh), a neurotransmitter which is then released into the synaptic cleft. To relay the nerve impulse, the neurotransmitter ACh goes and binds to the ACh receptors (AChR) present on the post-synaptic membrane of the other neuron. For a neuron to receive another impulse, ACh should be in low concentration at the cleft and must be released from the ACh receptor. Here, AChE, also located on the post-synaptic membrane, terminates the signal by hydrolyzing ACh and the liberated choline is taken back by the pre-synaptic neuron to recycle. This uptake, reuptake, and re-synthesis of ACh is responsible for neurotransmission at the neuromuscular junction. However, inhibition of AChE leads to accumulation of ACh in the synaptic cleft resulting in impaired neurotransmission thus succumbing mosquito to death^5^. Insecticides belonging to class of organophosphates and carbamates functions by efficiently inhibiting AChE^6^. However, at present, the drawback for many of such insecticides is their indiscriminate rates of application, environmental hazards and evolution of resistance amongst mosquito vectors, thereby paving the way for development of safer, non-toxic and environment friendly alternatives. For this, researchers have thought to make use of phytochemicals, plant extracts and essential oils to check their potency as an alternative to chemical insecticides. There are sizeable evidences for natural compounds from plant and their extracts to induce mortality in various vectors which is evident from the recent reviews published by Gajger and Dar^7^ while Shaalan and colleagues have specifically described potential phytochemicals that possess mosquitocidal potential^8^. However, the mode of action by which these phytochemicals induce mortality is underdetermined^9^. Advancement in data curation has made it feasible to screen thousands of compounds from such open access libraries to identify the lead molecule for any target protein^10–13^. This is where the rationale of the research represented in this experimental work is laid upon, that is to identify the natural molecule from an open access phytochemical library that can bind to AChE, inhibit its function, and induce mortality in mosquito by strategic use of in silico, in vitro and in vivo studies.

**Figure 1:**
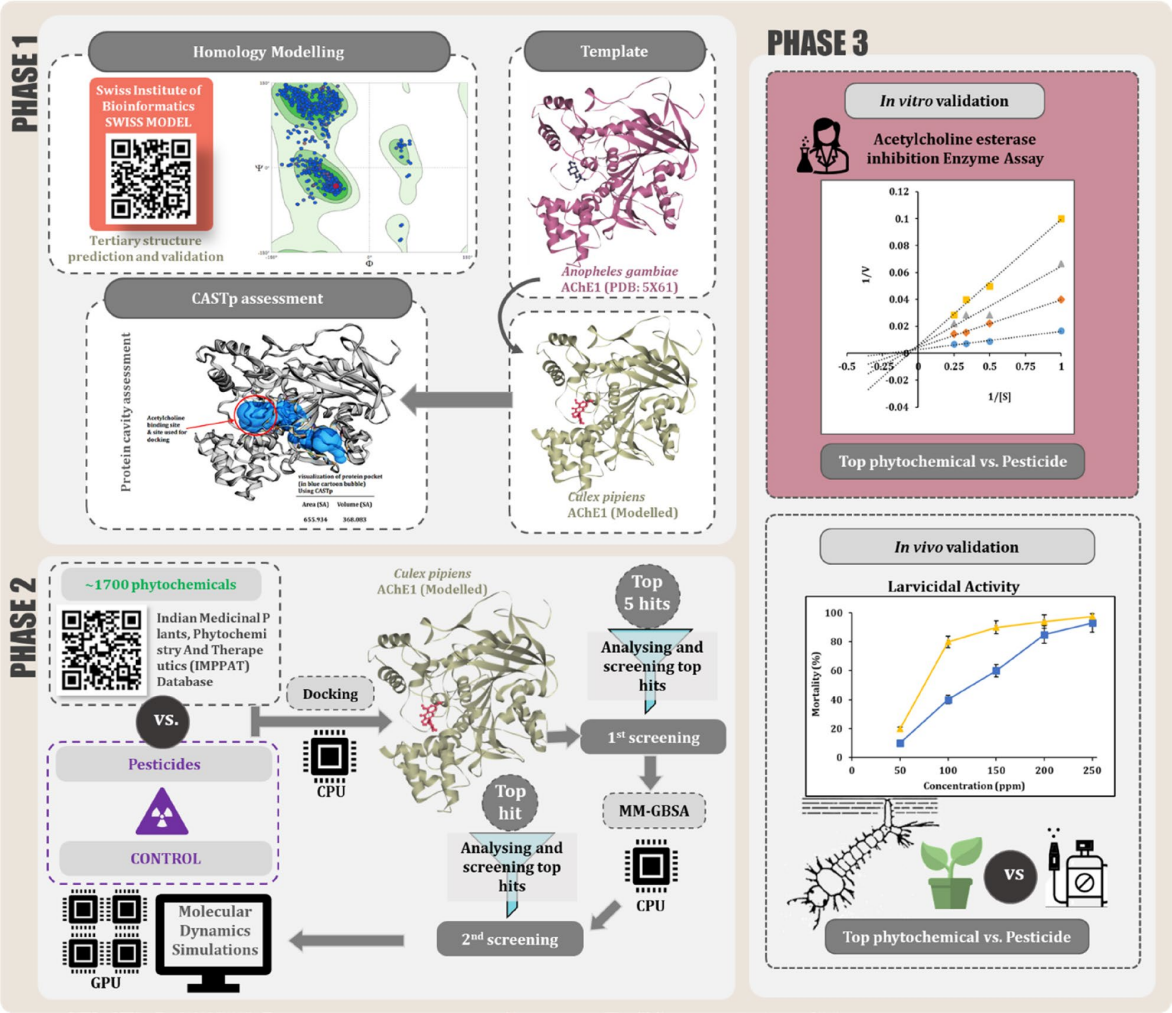
Overview of the workflow.

Entire work is divided into three phases as depicted in Fig. 1. The first phase of this research deals with an in silico structure prediction of *Cx. pipiens* AChE1 protein as the crystallized 3D model of *Cx. pipiens* AChE protein is unavailable at protein structure repositories. The second phase of this study comprises of in silico study for identifying the phytochemical that can best bind at the catalytic site of the protein to inhibit its function. For this, ~ 1700 naturally occurring phytochemicals from a curated database ‘IMMPAT’^14^ were screened by making use of molecular docking. Molecular docking only predicts the type of interaction that may occur between protein and ligand for a given pose, but the strength of the interaction is not predicted. Therefore, to validate result of docking assessment, MM-GBSA analysis was performed to predict the Gibbs free energy change which reflects spontaneity of ligand receptor interaction. Further, Molecular Dynamics (MD) simulation was performed to study stability of best docked pose of ligand with respect to its interacting protein. The third phase deals with validating the identified in silico findings with in vitro and in vivo assays. AChE enzyme inhibition assay of transmission vector *Cx. pipiens* was carried out using the lead phytochemical as an inhibitor at the larval stage of its life cycle. Subsequent larval mortality caused by the inhibition of insect AChE was evaluated using in vivo larvicidal bioassay. This study is conclusively able to determine a plant secondary metabolite larvicide as an alternative to organophosphates and carbamates following the similar course of inhibitory action on protein AChE.

## Result

### Structure prediction assessment

**Figure 2:**
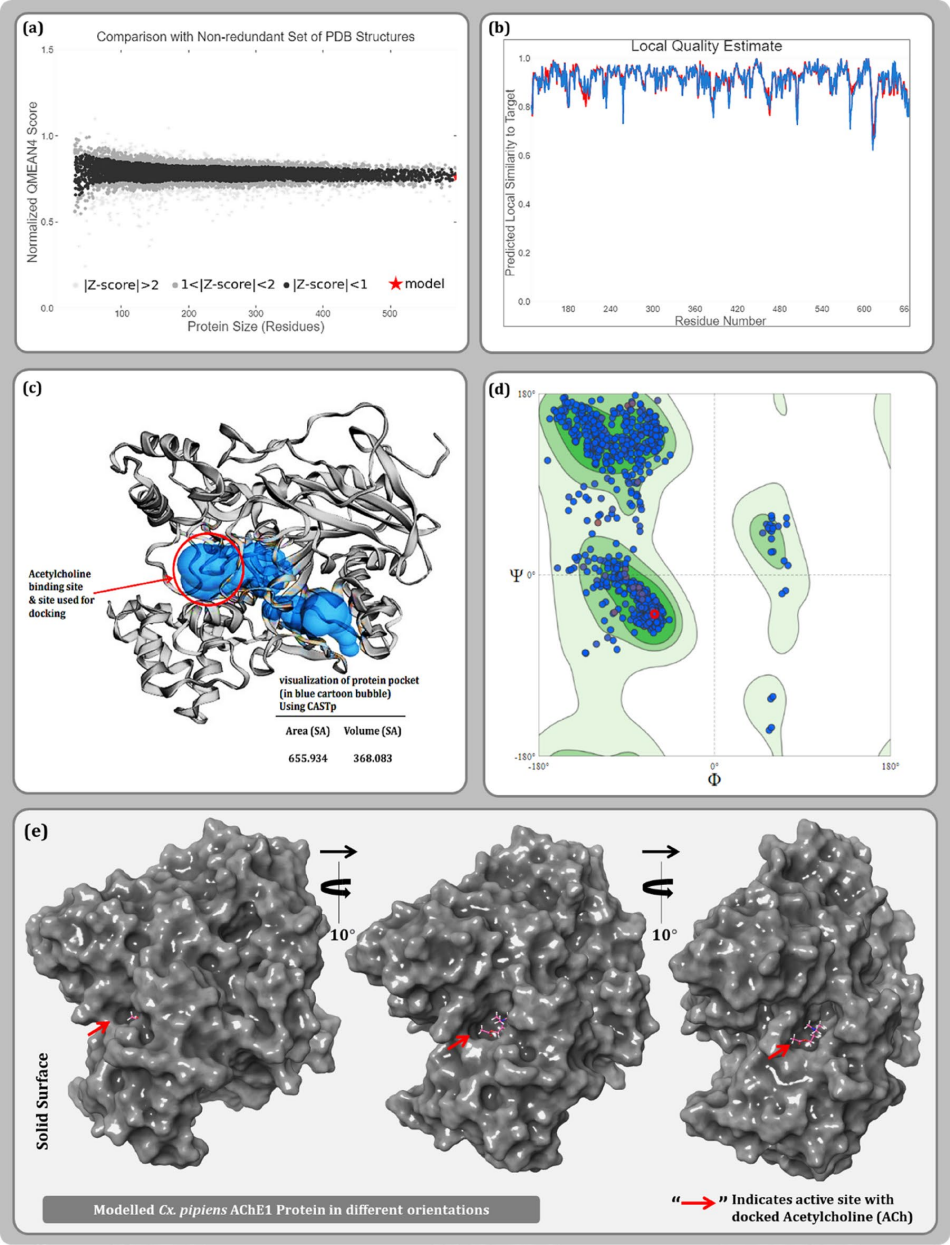
Quality estimate parameters for modelled AChE1 protein (**a**) comparison with non-redundant set of PDB structures (**b**) local model quality estimate (**c**) binding pocket identification of modelled protein (**d**) Ramachandran plot and (**e**) Modelled AChE1 in surface representation with docked ACh.

**Figure 3:**
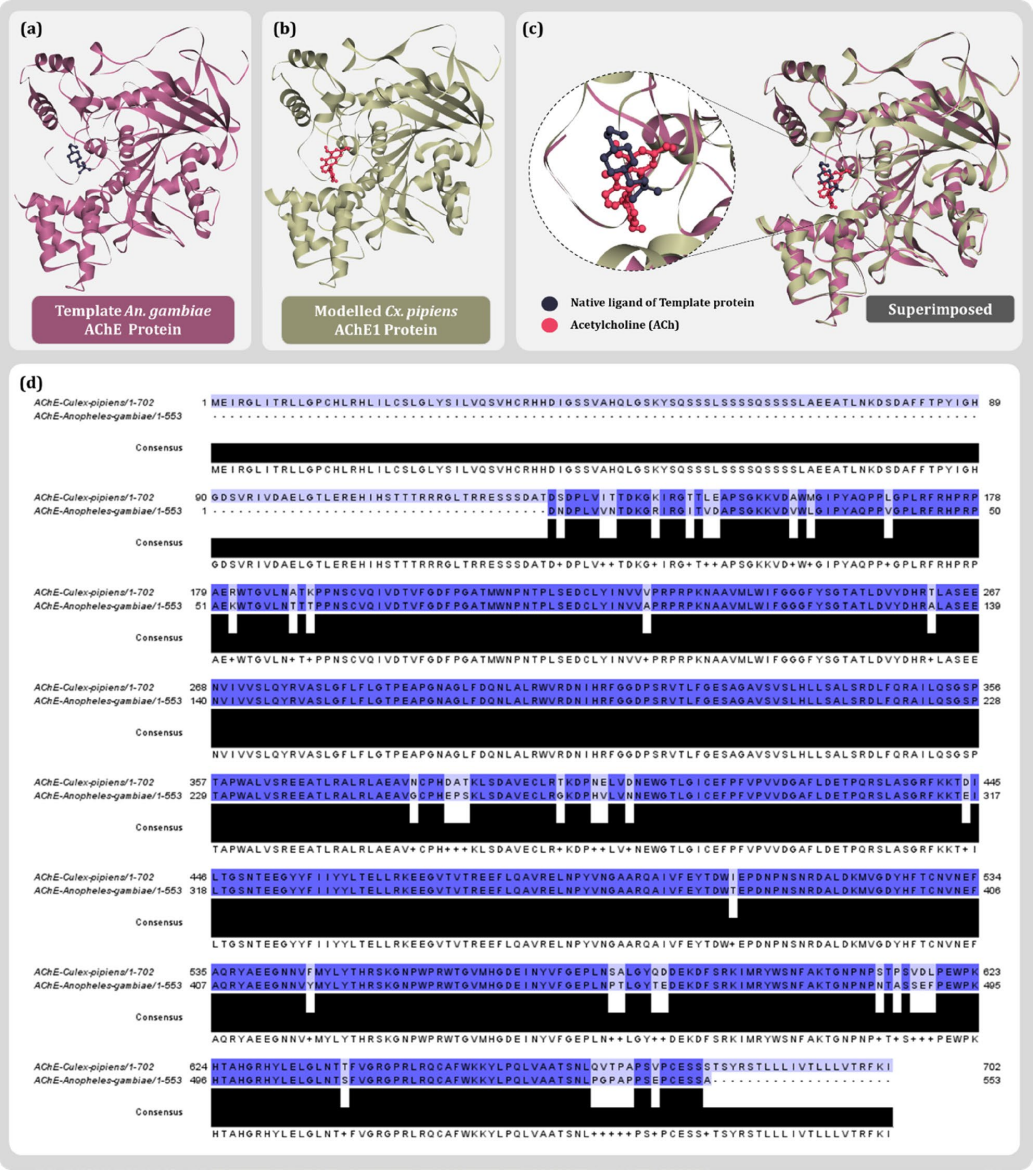
(**a**) *An. gambiae* AChE with co-crystallised ligand (PDB ID:5X61) (**b**) modelled *Cx. pipiens* AChE1 with biological ligand acetylcholine (**c**) Superimposed Model-Template alignment (**d**) Target-template pairwise sequence alignment of AChE1 protein of *Cx. pipiens* and AChE of *An. gambiae*.

The model for *Cx. pipiens* AChE1 was built using the template protein of malaria mosquito vector *Anopheles gambiae* (PDB ID: 5X61, Chain B), with 99% query coverage and 92.36% similarity. Upon successful model generation, quality check assessment was performed using various parameters as described in Fig. 2. The GMQE score ideally articulated between zero and one, is 0.65 for this target-template indicating a good structural reliability. Another parameter, QMEAN Z-scores closer to zero indicates good agreement between the model structure and experimental structures of similar size. Scores of − 4.0 or below is an indication of models with low quality. The QMEAN Z-score for the proposed model is − 0.07 indicating good model quality. MolProbity results of Ramachandran plot indicated 94.77% favored residues accounting for 0.45% outlier for the built model. Protein cavity assessment using CASTp 3.0 server, revealed the hydrophobic cavity of the modelled protein and a probable ligand binding cleft to be of volume 368.083 Å^3^ and surface area of 655.93 Å^2^ constituting following amino acid residues: Ile:70, Val:71, Tyr:121, Gln:197, Ile:198, Val:199, Asp:200, Thr:201, Val:202, Trp:212, Asn:213, Pro:214, Trp:242, Phe:244, Gly:245, Gly:246, Gly:247, Tyr:249, Ser:250, Gly:251, Leu:255, Tyr:258, Trp:280, Ile:285, Cys:286 Phe:288, Glu:326, Ser:327, Tyr:332, Tyr:333, Phe:329, Trp:360, Trp:408 and Gly:412 (Fig. 2d). Ensuring the amino acid residues by this means in the active site of the modelled protein, the co-ordinates were noted which were further used for preparing the grid during molecular docking. For validation of the active site, both the proteins i.e., template protein (Fig. 3a) and the modelled AChE1 (Fig. 3b) were superimposed as shown in Fig. 3c, to affirm an identical ligand binding cleft for both the proteins. Further, target-template similarity of sequence of AChE1 protein of *Cx. pipiens* and *An. gambiae* (Fig. 3d) were aligned with Multiple Sequence Alignment (MSA) using Clustal Omega^15^. Hence, the model was repeatedly validated to be of a good quality and was used further for in silico studies as shown in Fig. 3.

### Molecular docking assessment

**Figure 4:**
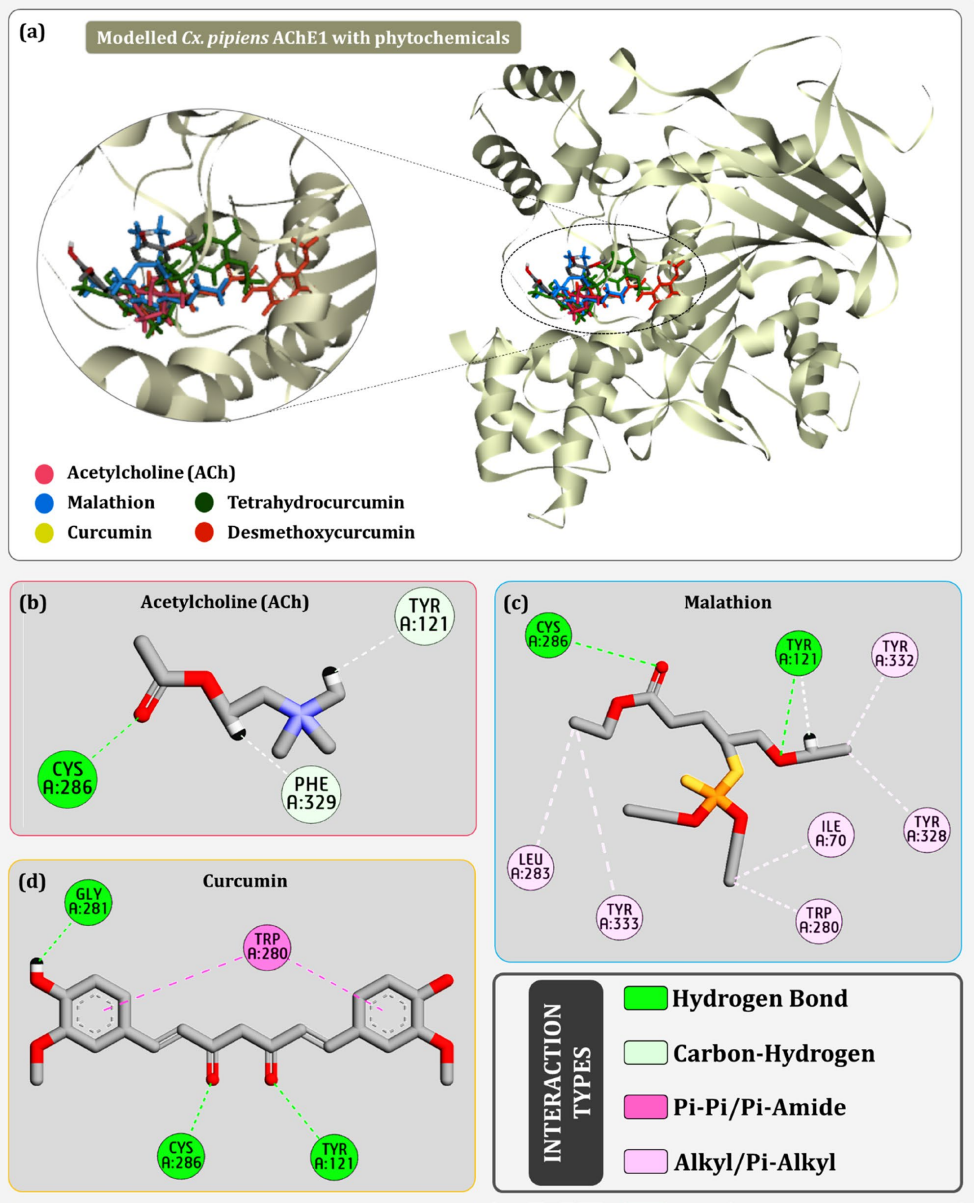
Protein–Ligand interaction profile (**a**) of all top hit compounds docked on modelled AChE1 (**b**) best docked pose conformation of acetylcholine (**c**) best docked pose conformation of malathion (**d**) best docked pose conformation of curcumin.

A rigorous computational virtual screening workflow was executed using ~ 1700 phytochemicals from IMMPAT database. Completion of the three precision levels of HTVS, SP and XP resulted in filtering top five lead compounds; curcumin, tetrahydrocurcumin, desmethoxycurcumin, bisdemethoxycurcumin and ar-turmerone as shown in Fig. 4a. Curcumin showed the most efficient binding with the binding energy of − 10.21 kcal/mol, followed by − 8.47 kcal/mol for tetrahydrocurcumin, − 8.01 kcal/mol for desmethoxycurcumin, − 7.42 kcal/mol for bisdemethoxycurcumin and − 7.21 kcal/mol for ar-turmerone. The interacting residues for each of these top hits are represented in Table 1. The XP docking of the modelled protein was also performed with reference biological ligand ACh and control insecticide malathion. It was found that both these ligands showed even lesser binding energy then the lowest ranked phytochemical in Table 1. The native ligand ACh forms a hydrogen bond with Cys:286 (Fig. 4b). The insecticide malathion also seems to make interaction with Cys:286 and Tyr:121 (Fig. 4c). Suggesting that Cys at position 286 is a crucial amino acid of the active cleft which is essential to be recruited for effective interaction of the ligand with the protein. Curcumin while interacting with AChE forms a total of three hydrogen bonds by interacting with Gly:281, Cys:286 and Tyr:121 (Fig. 4d).

**Table 1:** Docking score**,** binding energies and amino acid interaction profile obtained by performing molecular docking.

Ligand	Docking score	Binding energy (kcal/mol)	Compound ranking based on binding energy	Amino acid interactions
Acetylcholine	− 4.70	− 30.924	Biological substrate (Control)	TYR:121 CYS:286 PHE:329
Malathion	− 5.42	− 35.429	Known synthetic inhibitor	ILE: 70 TYR:121 TRP:280 LEU:283 CYS:286 TYR:328 TYR:332 TYR:333
Curcumin	− 10.21	− 55.558	1	TYR:121 CYS:286 GLY:281 TRP:280
Tetrahydrocurcumin	− 8.47	− 38.357	2	ILE:70 ASP:72 TYR:121 LEU:283 GLU:287
Desmethoxycurcumin	− 8.01	− 33.375	3	TRP:84 TYR:130 GLU:198 GLY:284 CYS:286 PHE:288
Bisdemethoxycurcumin	− 7.42	− 30.261	4	TYR:121 TRP:283 CYS:286 TYR:333
Ar-turmerone	− 7.21	− 30.131	5	ILE:70 TYR:121 TRP:280 CYS:286 TYR:328 PHE:329 TYR:332 TYR:333

### Filtering ligands through MM-GBSA calculation

The spontaneity of the ligands interacting with protein can be judged by free energy change using MM-GBSA calculations. The ΔGBind energy conveyed from MM-GBSA assessment is based on the bond-interaction advancement and often suggests the stability of the protein–ligand interaction. In general, lowest negative energies represents the higher stability the protein–ligand docked complex. The fidelity of the protein–ligand interaction was taken as a basis to identify a single best lead molecule out of the top five screened ligands from the database. The MM-GBSA profiles of all the top 5 lead compounds in comparison with reference control ACh and malathion are represented in Table 2.

**Table 2:** MM/GBSA profile for selected ligands with *Cx. pipiens* modelled AChE1 protein.

Ligand	ΔGBind (kcal/mol)	ΔGCoulomb (kcal/mol)	ΔGHbond (kcal/mol)	ΔGLipo (kcal/mol)	ΔGPacking (kcal/mol)	ΔGvdW (kcal/mol)
Acetylcholine	− 31.576	− 23.820	1.357	− 0.559	− 8.414	0
Malathion	− 39.958	− 17.339	2.967	− 0.810	− 18.239	− 2.254
Curcumin	− 62.283	− 18.466	5.427	− 1.692	− 29.856	− 2.060
Tetrahydrocurcumin	− 54.108	− 10.053	4.308	− 1.126	− 25.383	− 3.091
Desmethoxycurcumin	− 51.629	− 15.973	− 0.844	− 1.824	− 24.906	− 4.620
Bisdemethoxycurcumin	− 45.488	− 19.162	0.0889	− 1.0829	− 14.309	− 5.036
Ar-turmerone	− 43.520	− 21.831	0.0109	− 1.122	− 15.858	− 5.100

*ΔGBind* = binding energy, *ΔGCoulomb* = Coulomb energy, *ΔGHbond* = hydrogen-bonding correction, *ΔGLipo* = lipophilic energy, *ΔGPacking* = Pi-Pi packing correction, *ΔGvdW* = Van der Waals energy.

The interaction of ACh with modelled AChE1 as modelled AChE1-ACh complex occurred spontaneously as the ΔGBind is − 31.576 kcal/mol. However, the native biological ligand is a neurotransmitter, and the basis of neurotransmission is to carry out impulse at a rapid speed to generate either inhibitory or excitatory action at the neuromuscular junction, this justifies the lesser spontaneity of the ΔGBind of modelled AChE1-ACh complex with respect to ΔGBind value of the inhibitors that do require a greater binding affinity to carry out inhibitory action on the protein. Ranking in order of binding energy change from poor to best from all the screened inhibitors, malathion as the modelled AChE1-malathion complex has the ΔGBind value of − 39.958 kcal/mol, followed by docked complex of modelled AChE1-ar-turmerone with ΔGBind of − 43.520 kcal/mol. For modelled AChE1-bisdemethoxycurcumin docked complex, ΔGBind value is − 45.488 kcal/mol and for modelled AChE1-desmethoxycurcumin docked complex the value is − 51.629 kcal/mol, while the ΔGBind value for modelled AChE1 with tetrahydrocurcumin is − 54.108 kcal/mol. The best ligands to interact is curcumin in the docked complex of modelled AChE1-curcumin having the ΔGBind value of − 62.283 kcal/mol. Other parameters like Coulomb energy (ΔGCoulomb), Hydrogen-bonding correction (ΔGHbond), Lipophilic energy (ΔGLipo), pi-pi packing correction (ΔGPacking) and Van der Waals energy (ΔGvdW) comprises the total energy change of the system ΔGBind as observed in Table 2. All these parameters conclusively help to identify curcumin as a lead compound from all the ~ 1700 phytochemicals screened from the IMMPAT database to be the inhibitor of modelled *Cx pipiens* AChE1 and this was further validated through MD simulations.

### In silico validation from MD simulation

Curcumin was identified to be the best lead amongst all the screened phytochemicals as it was producing consistent results in parameters like docking score, protein–ligand interactions, and MM-GBSA calculations. To validate this further, MD simulation of the best docked pose of curcumin was performed. The docked complexes of modelled AChE1-ACh, modelled AChE1-malathion and modelled AChE1-curcumin were subjected to a 50 ns MD simulation, where the simulation profile for docked complex of ACh and malathion was taken as reference set. Once the simulations were performed, the Root Mean Square Deviation (RMSD), Root Means Square Fluctuation (RMSF) and protein–ligand contact profiles for all the frames of trajectory were calculated.

**Figure 5:**
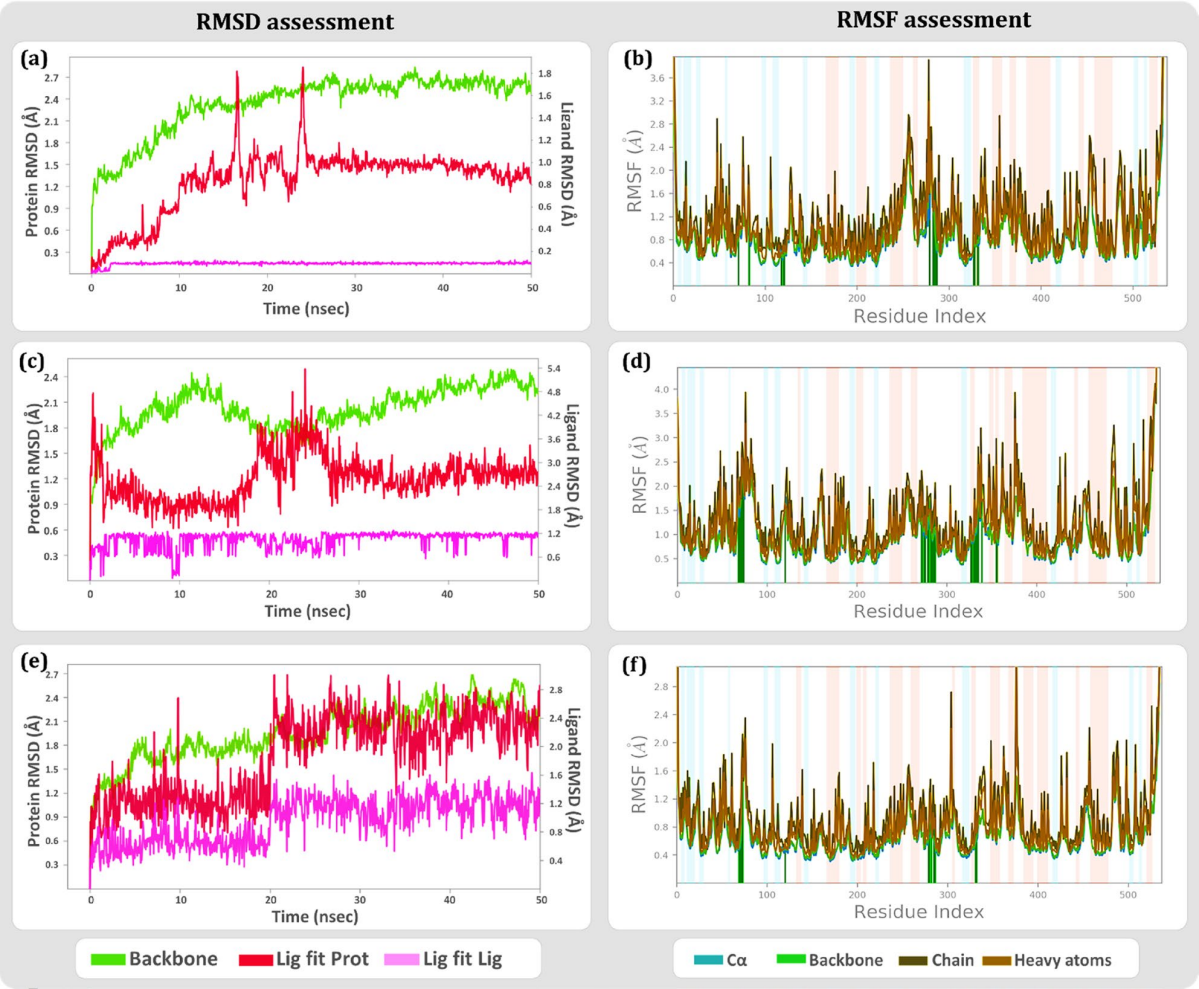
Protein–ligand interaction root-mean-square-deviation (RMSD) and root-mean-square-fluctuation (RMSF) profile of (**a**) AChE1-ACh complex RMSD (**b**) AChE1-ACh complex RMSF (**c**) AChE1-malathion complex RMSD (**d**) AChE1-malathion complex RMSF (**e**) AChE1-curcumin complex RMSD (**f**) AChE1-curcumin complex RMSF.

The plots in Fig. 5 depicts the RMSD movements (left *Y*-axis) in the portions of the protein. The RMSD assessment must measure the normal change in particle dislodging for a certain portion of frames as for a reference constant frame that is established for each frame of the trajectory. Here, typically the first frame of the ligand and protein for modelled AChE1-ACh, modelled AChE1-malathion and modelled AChE1-curcumin in the complex is used as the reference frame, and the movement for this initial alignment is then simulated during the entire 50 ns simulation. Change in displacement of the order of 1–3 Å are perfectly acceptable for small, globular proteins. In the case of modelled AChE1, its interaction with biological ligand ACh shows a RMSD value of 2.7 Å (Fig. 5a), while in terms of the inhibitor’s, curcumin (Fig. 5e) depicts a rather stabilised and equilibrated value of 1.8 Å with respect to malathion’s 2.4 Å RMSD (Fig. 5c). Another parameter on the same graph is Ligand RMSD (right Y-axis) which indicates how stable the ligand is with respect to the protein at its binding site. For the docked complex with ACh the ligand RMSD is 1.8 Å, for malathion is 5.4 Å and for curcumin is 2.25 Å. Generally, it is observed that if the ligand RMSD values are much greater than the protein RMSD values, the ligand has most certainly diffused away from its initial binding site on the protein. Seeing this value, it can be stated that malathion is likely to dislodge from the active cleft in comparison with curcumin, making curcumin a stable inhibitor to bind the modelled protein.

Further, analysis of RMSF plot for the docked complexes is useful for characterizing local changes along the protein chain and peaks on this plot indicate areas of the protein that fluctuate the most during the simulation. The large presence of tails of N and C terminals showcase higher fluctuations in the corresponding peaks while secondary structure elements like α helices and β strands are usually more rigid and show less fluctuation. In this case, the docked complexes of modelled AChE1-ACh (Fig. 5b), modelled AChE1-malathion (Fig. 5d), and modelled AChE1-curcumin (Fig. 5f) depict protein interaction with the ligands and trends of RMSF correspond similarly for ACh and malathion while for curcumin the value suggests better stability. This is because the binding of curcumin at the active cleft has allowed α helices and β strands to become rigid and thereby stabilising the local changes along the protein chain.

**Figure 6:**
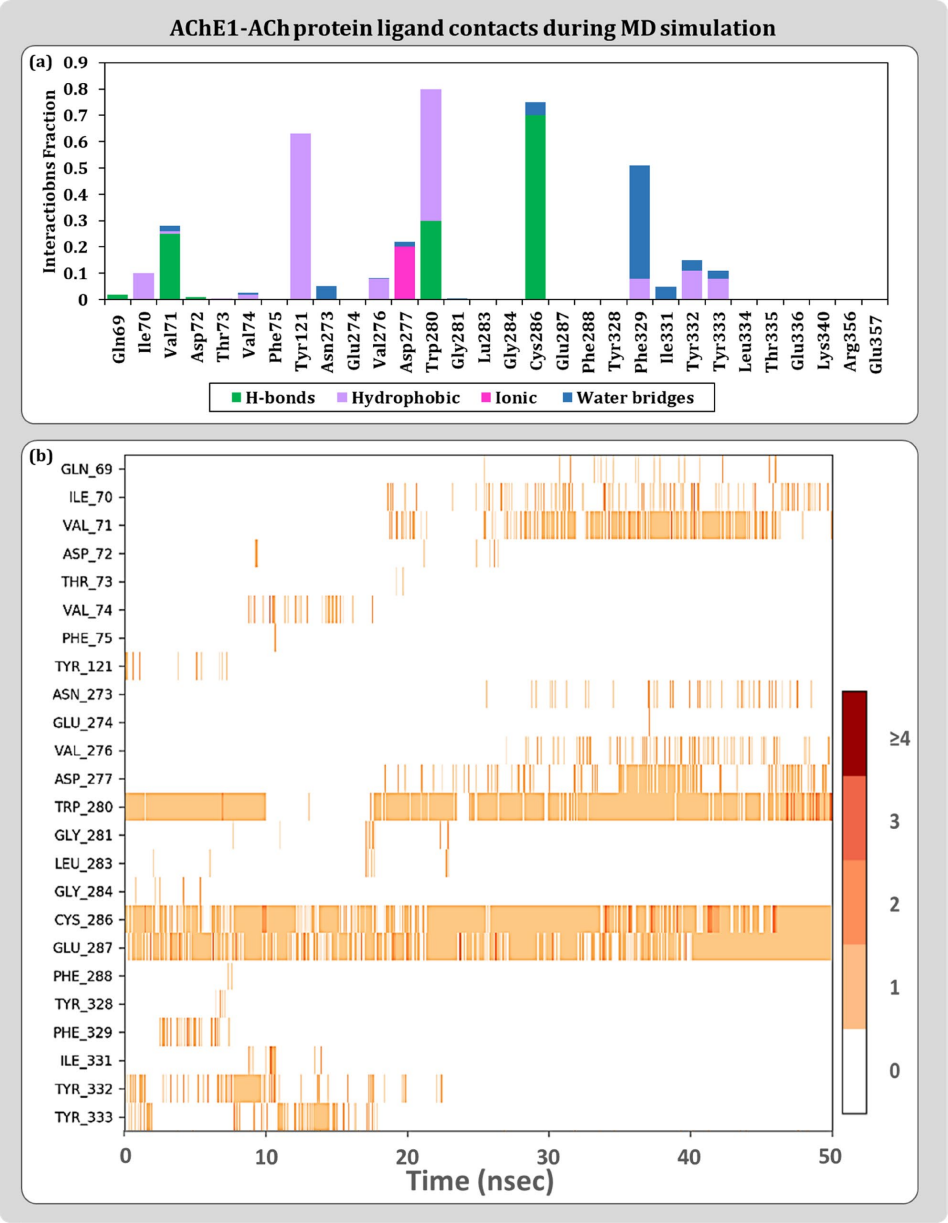
Protein–Ligand interaction profile for AChE1-ACh complex (**a**) Ligand contact points (**b**) Timeline representation.

**Figure 7:**
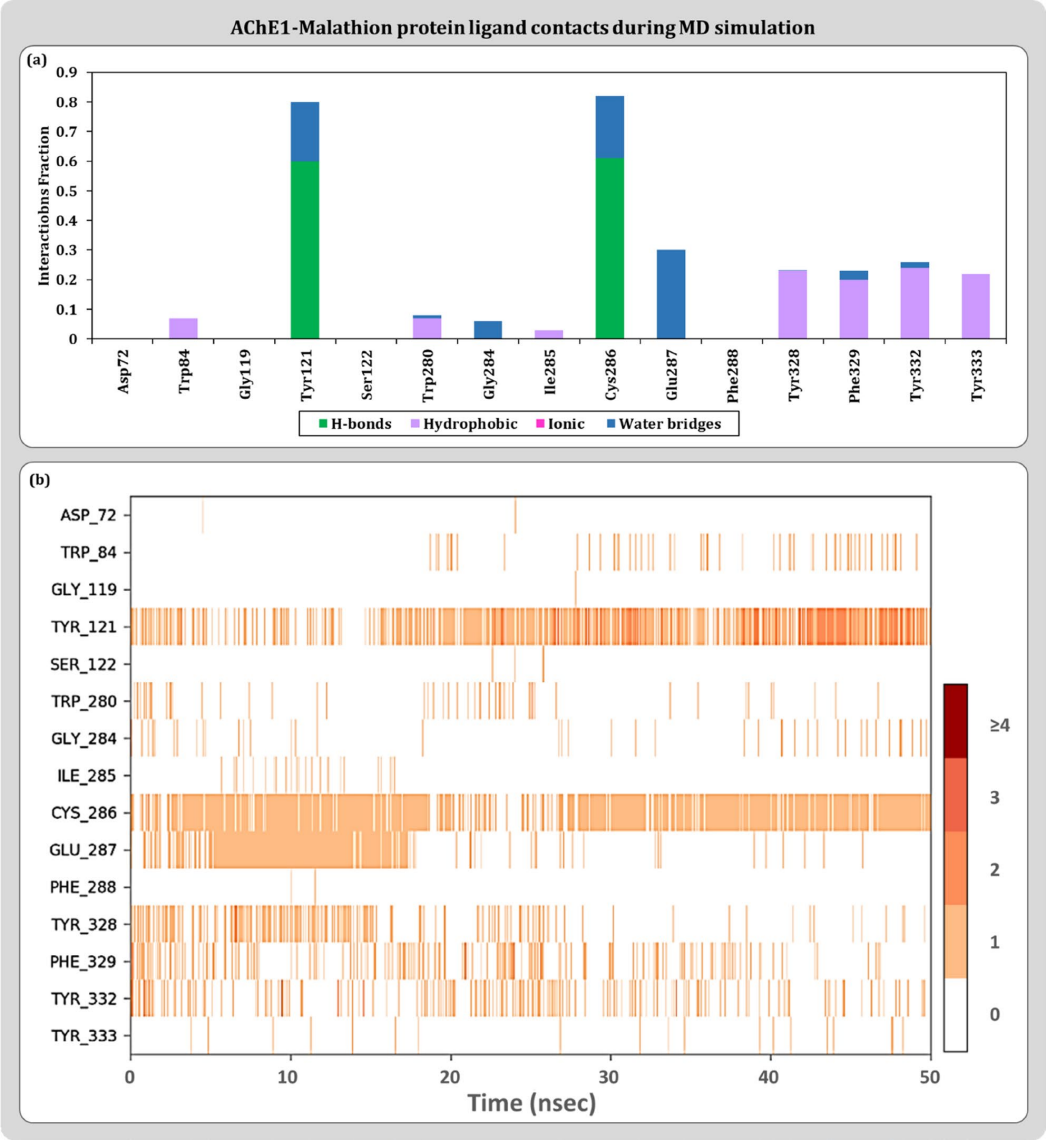
Protein–Ligand interaction profile for AChE1-malathion complex (**a**) Ligand contact points (**b**) Timeline representation.

**Figure 8:**
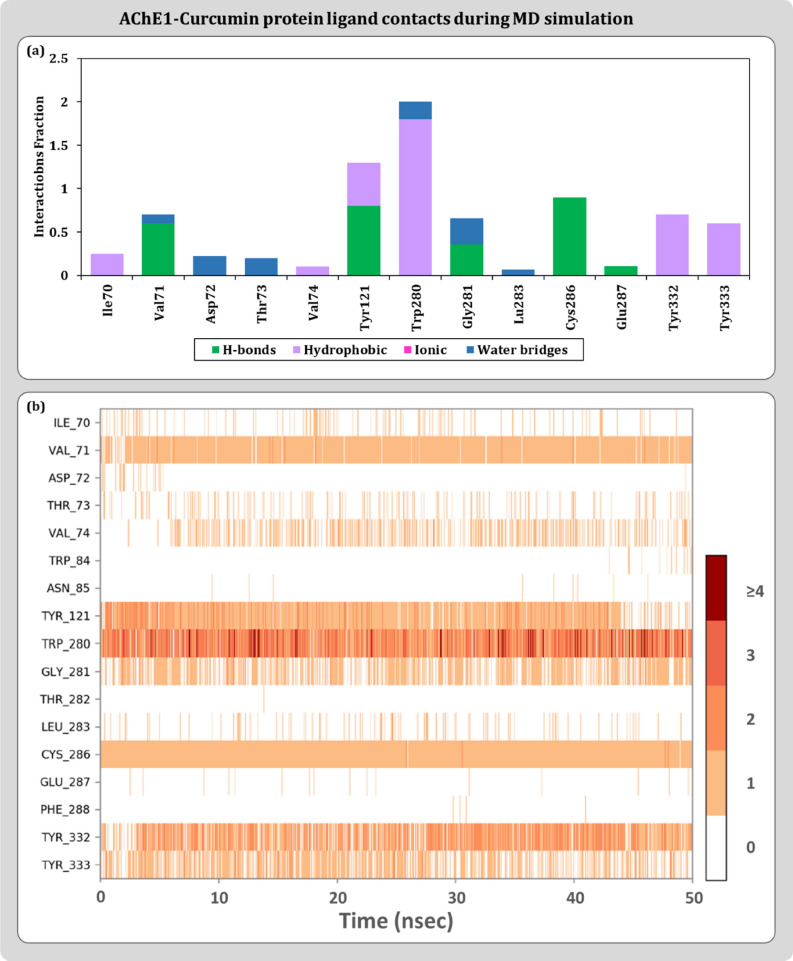
Protein–Ligand interaction profile for AChE1-curcumin (**a**) Ligand contact points (**b**) Timeline representation.

Protein–ligand contact profiles are represented as stacked bar charts type and timeline representation. The stacked bar charts are normalized by representing the percentages in decimals. For a timeline representation, the top panel shows the total number of specific contacts the protein makes with the ligand over the course of the trajectory. The bottom panel shows which residues interact with the ligand in each trajectory frame. Some residues make more than one specific contact with the ligand that are represented by a darker shade of orange, according to the scale to the right of the plot. For modelled AChE1-ACh, the common interactions include amino acids like Val:71, Tyr:121, Val:276, Asp:277, Trp:280, Cys:286, Glu:287 and Phe:329 as contact residues as shown in Fig. 6. A portrayal of protein–ligand interactions for modelled AChE-malathion suggests involvement of Tyr:121, Gly:119, Cys:286, Glu:287, Phe:329 and Tyr:333 amino acid residues as describes in Fig. 7. For curcumin, interaction profile includes Val:71, Tyr:121, Trp:280, Gly:281, Cys:286, Glu:287, Tyr:332 and Tyr:333 as shown in Fig. 8. Hydrogen bonds are very much crucial in determining the specificity and stability of the contact and in curcumin is able to engage 100% of 50 ns simulation. These MD results also helps to conclude the reliability of the docked pose of curcumin with AChE1 in comparison to malathion.

### In vivo larval mortality assessment

**Figure 9:**
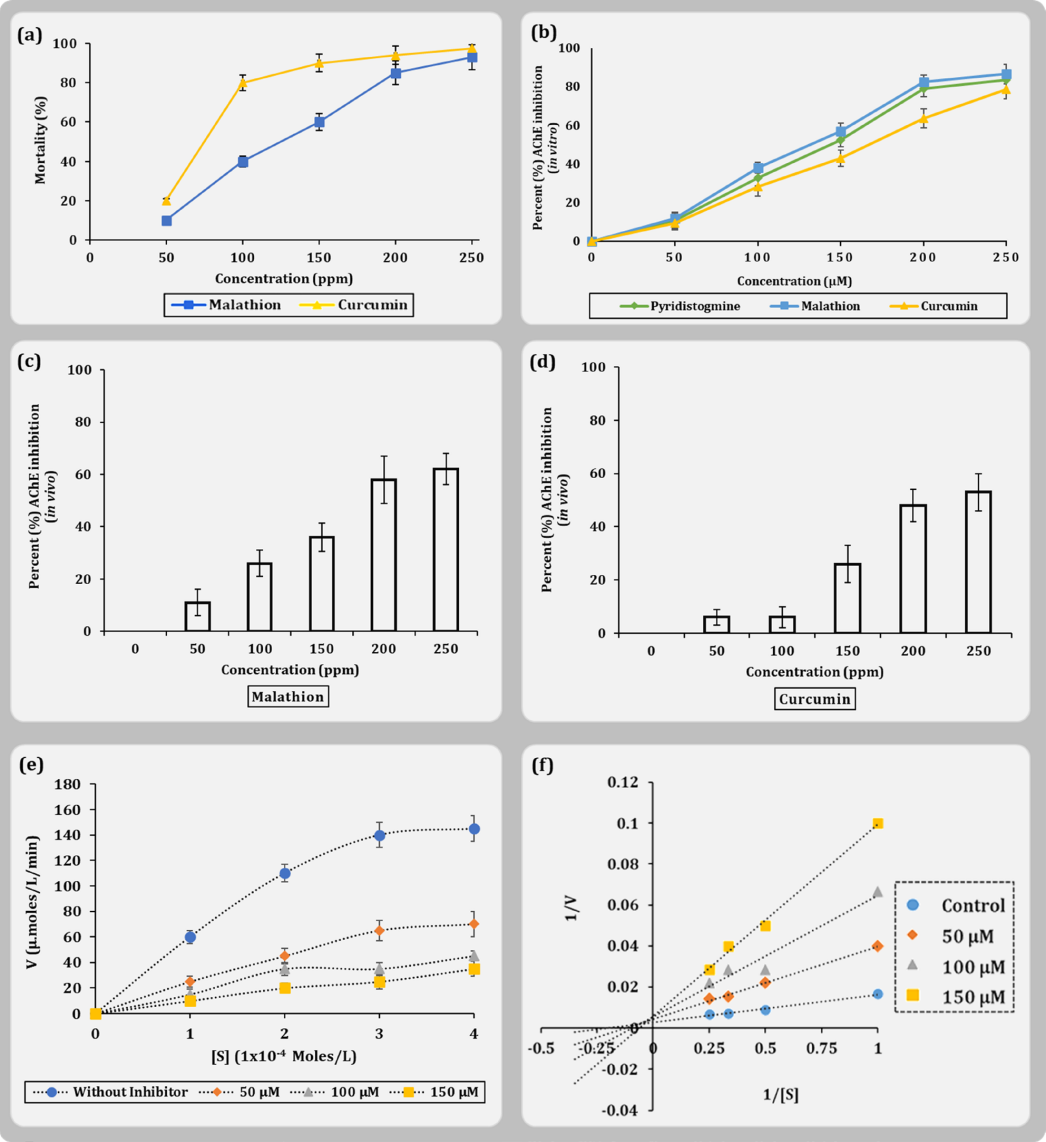
Result demonstration for in vivo and in vitro bioassay (**a**) Dose response curve for malathion and curcumin (**b**) in vitro AChE inhibition activity by pyridostigmine, malathion and curcumin, (**c**) in vivo AChE inhibition by malathion, (**d**) in vivo AChE inhibition by curcumin (**e**) Michaelis–Menten plot with different inhibitor (curcumin) concentration (for **a**–**e**; n = 4, error bars, standard error of mean) (**f**) Lineweaver-Burke plot representation of the MM equation.

In silico assessment showed curcumin to best interact with the AChE1 of *Cx. pipiens.* To affirm the claims drawn by the computational workflow, curcumin was subjected for two parallel assays. First, the in vivo insecticidal potential of commercially available pure curcumin was studied on the late 3rd and early 4th instar larvae of *Cx. pipiens* with keeping the chemical insecticide malathion as positive control. Second, in vitro AChE inhibition by curcumin was examined from the larval extract keeping two positive controls, (i) known AChE inhibitor pyridostigmine bromide and (ii) malathion, known insecticide and AChE inhibitor. For the first set of experiment, it was observed that curcumin exhibited phenomenal larvicidal potentials by inducing ~ 80% of mortality, at the working concentration of 100 ppm and LC_50_ of 112 ppm (Fig. 9a). Moreover, at this same concentration, malathion could induce only about ~ 38% mortality. For malathion to induce ~ 80% mortality, the dosage of application was as high as 200 ppm. This reflects that, curcumin can serve as a potential larvicidal, if not for all insect but for *Cx. pipiens* larvae, and the potency to dosage ratio for inducing mortality was half to that obtained for malathion.

### In vitro and in vivo AChE inhibition assay

For the second set of study, to identify the mode of action of larval mortality, deals with the larval AChE inhibition. It was observed that curcumin could inhibit the enzyme AChE activity at par with the pyridostigmine bromide and malathion (Fig. 9b). It was observed that at 250 µM concentration, all the three compounds (curcumin, pyridostigmine and malathion) under study inhibited ~ 80% of AChE enzyme activity. The IC_50_ value so obtained for curcumin was 167.09 µM with R^2^ value 0.993, for malathion was 143.76 µM with R^2^ value 0.9784 and the IC_50_ value obtained for pyridostigmine bromide was 135.54 µM with R^2^ value 0.9767. This confirmed the predictions prior made by in silico studies that curcumin can interact with AChE and can inhibit its function. On affirming by in vitro studies, we performed the in vivo enzyme inhibition assay, where we found that AChE inhibition was observed when larvae were incubated for 30 min in solutions of malathion (Fig. 9c) and curcumin (Fig. 9d) in the concentrations ranging from 50 to 250 ppm. Identifying the nature of enzyme inhibition becomes the next important parameter to be accessed. It is therefore inevitable to understand that AChE enzyme inhibition by curcumin is competitive or non-competitive. For this, in vitro AChE inhibition enzyme assays were performed with varying concentrations of curcumin and the relation between substrate concentration to rate of enzyme activity was evaluated. The results of this study were plotted as an enzyme activity versus substrate concentration (Fig. 9e) and the same results were also plotted by constructing double reciprocal lineweaver–burke plot (Fig. 9f). Both these graphs help to identify the change in the K_M_ (Michaelis–Menten constant) of the enzyme reaction occurring in the presence of different concentrations of inhibitor (curcumin). Lineweaver–burke plot is more accurate in calculating the K_M_ of the enzyme reaction. In this plot the intersection of the negative extrapolation with the negative x-axis represents the ‘–(1/K_M_)’. From this graph it is observed that as the concentration of curcumin increases the ‘–(1/K_M_)’ becomes larger (Fig. 9e). Making the subject of the formula to ‘K_M_’, it is observed that K_M_ value increases with increasing the concentration of curcumin, which is a characteristic nature of competitive inhibition. Thus, from the entire study it can be well deduced that, curcumin exhibit larvicidal activity and can inhibit *Cx. pipiens* larval AChE in the competitive manner which ultimate serves as its mode of action for larvicidal activity.

## Discussion

World Health Organization, as per their latest publication entitled “Global vector control response 2017–2030” claimed that mosquito-borne diseases have spreaded all through the globe and affects 350 million people worldwide annually^1^. Of all the mosquitoes that contribute the spread of diseases, *Cx. pipiens* holds a significant share along with *An. gambiae*. In particular, *Cx. pipiens* are notable transporters of west nile infection, saint louis encephalitis infections, avian intestinal sickness, and filarial worms^3^.

Chemical pesticides such as organochlorine compounds, organophosphates, carbamates, pyrethroids and formamidines so categorized as second generation of chemical pesticides are cheap and are primarily used to curb seasonal outburst of mosquitoes by their use being as high as 1 million pounds a year globally^2^. Such overwhelming use of these chemicals comes with the cost. First, they enter the food chain by getting adsorbed on the edible plant parts like fruits and vegetables posing great health hazards for humans. Somehow for this reason humans shall not completely rely on chemical pesticides. Second, the progressive over exposure of chemical pesticide leads to evolutionary epigenetic adaptation in flies and mosquitoes leading to acquired resistance. The housefly strains (*Musca domestica*) serve as a classic example as they have developed resistance to virtually every insecticide used against them^16^. Last but not the least, their non-degrading nature leads to their bioaccumulation ultimately polluting agriculture soil which makes it important to develop new and safer alternatives.

While most of the carbamates and organophosphates available are AChE inhibitors like propoxur (Baygon), acephate, chlorpyrifos, bendiocarb, malathion, ethion, famphur, temephos abates or temephos sand granules^17^; DDT and pyrethroids like resmethrin, permethrin or deltamethrin are axonic excitotoxins which act by inhibiting the closure of voltage gated sodium channels in the axonal membranes^18^; juvenile hormone binding protein mimicking methoprene is used which is an insect growth regulator^19^; GABA receptors antagonists include avermectin, fipronil, chlordane heptachlor and phenyl pyrazole like compounds and many more^20^. These commercially available synthetic compounds possess exceptional long-term control over larvicidal activity. Of these, the most important activity is the inhibition of activity of protein AChE as it is a key enzyme responsible for terminating the nerve impulse transmission through synaptic pathway. The synaptic concentrations of ACh then builds up and hyperexcitation of the central nervous system occurs, composed of long discharges of action potentials arising from a single stimulus. The signs of intoxication include restlessness, hyperexcitability, tremors, convulsions, and paralysis. Organophosphorus and carbamate are observed to be AChE potent inhibitors^4^. Malathion is one of the most potent inhibitor of AChE and it is widely used to kill mosquitoes, and therefore due to its well-known inhibitory effect on AChE of mosquitoes^21^, we have chosen it as positive control for in silico, in vitro and in vivo studies. Moreover, Malathion is also reported to interact with AChE of human, this is because the enzyme AChE of pests like insects and mosquitos is identical to that of humans^22^. As most of the insecticides and larvicides resistance reports are about carbamates and organophosphates and we under this present study are trying to identify a particular phytochemical that can act as a potential substitute of these commercially available carbamates and organophosphates. Current research is built upon the rationale for identifying potential phytochemicals that inhibit ability to specifically bind to AChE1 protein and inhibit the same protein through competitive inhibition as its mode of action depicting larvicidal activity.

Many plants and their corresponding phytochemicals are known to potentially cause larval mortality. Shaalan et al. have vividly described at length the potential phytochemicals that possess mosquitocidal potential in their review^8^. Most of these phytochemicals also perform at par with the activity of commercially available synthetic larvicides, which leads to their promotion and suggestion for their use as herbal larvicide. Their failure in replacing the synthetic options happens so due to multiple underlying issues, like inability to prepare plant extracts or phytochemical extracts in bulk distribution quantities, insufficient application or larvicide or in excess of the recommended dosage. Another drawback is neglecting to study and gather experimental evidence regarding the mode of action of that particular phytochemical or plant extract in initiating larval mortality^8^. Under current study we have put our efforts to fill this knowledge gap. We have evaluated ~ 1700 compounds from IMPPAT database for their ability to interact with AChE, which is the mode of action of several synthetic pesticides. IMPPAT is the largest database on phytochemicals of Indian medicinal plants to date, and this resource is a culmination of the efforts to digitize the wealth of information contained within traditional Indian medicine. IMPPAT provides an integrated platform to apply cheminformatic approaches to accelerate natural product-based drug discovery. IMPPAT is also expected to enable application of system-level approaches towards future elucidation of mechanistic links between phytochemicals of Indian medicinal plants and their therapeutic action^14^. We made use of in silico methods, which is efficient and advance approach to identify compounds for specific targets, in this case AChE. Current study is not the only one of its kind, there are several researchers who have made use of in silico approach for identifying leads for AChE. Natural compound, 2,3-dimethylmaleic anhydride was previously reported to interact with AChE of cockroach and induce mortality^23^. Synthesis of chemical compounds as derivatives of oxamide and fumaramide were developed making use of similar in silico approach for the inhibition of human AChE and other identical target butyrylcholinesterase (BuChE) for treating Alzheimer’s disease^24^. We not only used routine protocols of docking, but we took a step beyond making our in silico studies more robust by involving MM-GBSA calculations and MD simulations. The sub-atomic mechanics energies joined with the Poisson–Boltzmann or summed up Born and surface territory continuum solvation commonly referred as MM-PBSA and MM-GBSA strategies are mainstream ways to deal with gauge the free energy of the ligands to macromolecular proteins. They are normally founded on sub-atomic elements simulations of the protein–ligand complex and in this way possess both precision and computational exertion between exact scoring and severe catalytic bother strategies^25^. The Prime module of Maestro used under present study, performs its own simulation based on the ‘best docked protein–ligand pose’ by using highly robust VSGB 2.0 energy model^26^. The MM-GBSA is applied to an enormous number of protein ligand interaction frameworks with tremendous success to validate the outcomes of molecular docking^13,27–30^. Further, the accuracy proposed by the docking assessment for interaction occurring between ligand and protein would actually translates into reality or not is evaluated robustly by MD simulations. The MD simulations will reassure the interaction length, interaction types occur as predicted by docking or not. Docking will only predict the type of interaction that may occur between protein with a ligand for a given pose, but the strength of the interaction is not predicted, this limitation of molecular docking is overwhelmed by MD simulations. Moreover, the stability of the best docked pose of ligand with respect to its interacting protein can also be evaluated over a course of time under MD simulations^12,31–34^. Further, validation of in silico studies were also done making use of in vitro AChE enzyme inhibition and then assessing the larvicidal activity of top hit using in vivo assay.

The study represents the phytochemicals, curcumin, tetrahydrocurcumin, desmethoxycurcumin, bisdemethoxycurcumin, and ar-turmerone to show potentials to interact with AChE. The second level of in silico assessment using MM-GBSA and MD simulations showed curcumin to the top lead. Later, as represented in the results section, curcumin’s efficacy to inhibit AChE was proved with in vitro assays and it also exhibited larvicidal activity on the larva of *Cx. pipiens*. There are reports suggesting curcumin to serve as insecticide^35,36^ but there are no reports suggesting its interaction with AChE of mosquitoes, serving as its mode of action of its insecticidal activity and that too specifically on dipteran’s belonging to genus *Culex*. Curcumin is the most abundantly found curcuminoid compound of turmeric (*Curcuma longa*), it is a phytochemical with bright yellow apparency. The second ranked compounds namely, tetrahydrocurcumin, desmethoxycurcumin, bisdemethoxycurcumin, and ar-turmerone are also reported to be found from the plant of turmeric. It is also reported that various derivatives of curcumin can inhibit AChE, Butyrylcholinesterase (BChE) and trypsin in mammals^37^. Curcumin, till date has been perceived as a wonder natural compound by possessing traits such as anti-inflammatory, anti-cancer, anti-Alzheimer, while also to be effective for nervous system, respiratory, cardiovascular, gastrointestinal, urogenital, and metabolic disorders. The beneficial traits of curcumin is phenomenally represented in the review article published by Salehi and colleagues in the year 2019^38^. To our interest, curcumin is previously reported to interact and inhibit the human AChE activity and therefore also known to serve as an anti-Alzheimer natural medicine^39–42^. There are reports for curcumin to interact with human AChE even with in silico assessments^43,44^. However, till date there are no reports suggesting the interaction of curcumin with the AChE of *Cx. pipiens* mosquito, and therefore this document serves as the first of its kind to represent the in silico, in vitro and in vivo assessment suggesting curcumin to interact with AChE of mosquito (*Cx. pipiens*) and inhibit its action and even serving as larvicide.

## Materials and methods

### Tertiary structure prediction through comparative homology modeling

AChE1 protein sequences (Accession Number: Q86GC8) of *Cx. pipiens* (house mosquito) was retrieved from UniProtKB (https://www.uniprot.org/uniprot/)^45^ in fasta format. SWISS-MODEL (http://swissmodel.expasy.org/)^46^, a protein modelling server, was used to predict the 3D structure for AChE1 of *Cx. pipiens.* The server developed homology models by performing a target-template sequence alignment using the BLASTp and HHBlits programs to search through template structures in Protein Data Bank (PDB)^47,48^ and SWISS-MODEL Template Library (SMTL) repositories. The model's quality was evaluated by using Z scoring functions of Global Model Quality Estimation (GMQE) and Qualitative Model Energy Analysis (QMEAN), which were exclusively developed for SWISS-MODEL^49^. MolProbity^50^ was used to develop Ramachandran plot and further evaluated the plot by determining the number of accepted and outlier amino acid residues of the proposed AChE1 protein model. The co-ordinates of ligand binding site on AChE1 modelled protein were determined using CASTp 3.0 server (Computed Atlas of Surface Topography of proteins)^51^ prior to molecular docking analysis.

### Protein–ligand docking

Prior to docking, the AChE1 modelled protein was prepared in the Protein preparation wizard of Maestro, Schrödinger Release 2021–2^52^. Protein preparation involves correcting charges, adding hydrogen bonds, assigning bond orders, filing missing loops and missing side chain residues to refine the 3D structure. After inspecting the protein reports, the structure was additionally optimised and minimised with default parameters under OPLS-2005 (Optimized Kanhesia for Liquid Simulations) force field^53–55^. Furthermore, Receptor Grid Generation wizard was used to generate the Glide docking grid box of size 10 Å × 10 Å × 10 Å at the centroid of the active cleft identified using CASTp 3.0 server at the co-ordinates − 79.11° on X axis, − 21.52° on Y axis and 95.70° on Z axis.

All the test ligands were retrieved in SDF format from Indian Medicinal Plants, Phytochemistry And Therapeutics (IMPPAT), a curated database which has been constructed via literature mining and manual curation from scientific literature on Indian medicinal plants^56^. 3D conformations for the biological substrate acetylcholine (ACh) (CID:187) and known chemical inhibitor malathion (CID:4004) were also retrieved in SDF format from PubChem^57^. LigPrep wizard of Schrödinger Release 2021-2 was used to prepare and minimize the ligands with Epik^58,59^ at a physiological pH of 7.4 unit under OPLS-2005 force field. The output files prepared during ligand minimization were used for molecular docking.

Molecular docking was carried out in three phases: (a) High throughput virtual screening (HTVS), (b) Standard Precision (SP), and (c) Extra Precision (XP) using the virtual screening workflow of Glide module of Schrödinger Release 2021-2. HTVS allows minimum torsional refinement for the docked poses making the process overall rapid. While SP and XP enforce higher torsional refinement to pass through the docking funnel. XP also employs a more sophisticated scoring function than SP for protein–ligand shape complementarity making this docking process robust. At each level of filteration, top 8% phytochemicals were screened from HTVS, SP and to XP. Out of all ~ 1700 phytochemicals, the top five screened lead compounds were selected following virtual screening based on docking score range (− 11.00 to − 7.00 kcal/mol).

### End-point binding free energy change calculation

Binding energies were computed using the Molecular Mechanics-Generalized Born Surface Area (MM-GBSA) method^25,60,61^. Application of this calculation is particularly important to determine binding free energy of biomolecular complexes like protein–ligand complex. The binding free energy was calculated according to the following equations:

1 $$ \Delta GBind = \Delta H - T\Delta S \approx \Delta EMM + \Delta GSolv - T\Delta S $$

where, ΔGBind is the free energy of the system resulting from the sum of the molecular mechanics energy (ΔEMM), solvation free energy (ΔGSolv), and entropy (− TΔS). MM-GBSA calculation was performed using the Prime module of Schrödinger Release 2021–2^62,63^. ΔGBind is the binding free energy calculated as per MM-GBSA. As the MM-GBSA binding energies are approximate free energies of binding, a more negative value indicates stronger binding and therefore ΔGBind of MM-GBSA is used to estimate relative binding affinity for a list of ligands (reported in kcal/mol). In brief, ΔGBind is the Gibbs free energy, that is residual enthalpy after subtracting entropy. The Prime module of Maestro used under present study, performs its own simulation based on the ‘best docked protein–ligand pose’ by using highly robust VSGB 2.0 energy mode.

### In silico validation through molecular dynamics (MD) simulation

Systems like protein–ligand complexes are dynamic in nature and therefore analyzing their motions at the molecular and atomistic level using MD simulation is essential in understanding the key physicochemical phenomena. Three set of simulations for AChE1-ligand complex were performed using Desmond package^34^ of Schrödinger Release 2018–4 for a period of 50 ns each. The first set involves the best docked complex of AChE1-ACh, the second set involves the docked complex of AChE1-lead phytochemical from IMPPAT database, and the final set was of the known inhibitor AChE1-malathion docked complex, taken as control.

Initially the energy minimization of protein ligand complex was performed using OPLS-2005 force field, after which the system was build using TIP3P solvent model which specifies a 3-site rigid water molecule with charges and Lennard–Jones parameters assigned to each of the 3 atoms. Periodic boundary conditions (PBC) were setup by selecting the orthorhombic shape simulation box fitting the protein ligand complex with having 15 Å buffer space around the periphery of the protein. Followed by neutralisation with placement of Na^+^ ions and salt concentration of 0.15 M Na^+^ and Cl^-^ counter ions to simulate the background salt and physiological conditions using OPLS-2005 force fields. Once the system gets incorporated, MD simulation was performed with NPT (constant Number of particles, Pressure, and Temperature) ensemble with 300 K temperature and 1.013 bar atomic pressure and default surface tension using Smooth Particle Mesh Ewald (PME) method to neutralise the electrostatic interactions^64^. For the simulation time of 50 ns, the energy recording interval was set at 1.2 ps and 1000 snapshots of the simulation trajectories were recorded. On completion of simulation, Desmond is integrated with a Simulation Interaction Diagram wizard which helps in analyzing the results for simulated trajectories.

### Mosquito rearing

Test organism *Cx. pipiens* eggs were obtained from City Civic center of Ahmedabad Municipal Corporation (AMC) Gujarat, India. The filter paper containing the mosquito eggs was placed in a plastic tray with 100 ml distilled water and allowed to hatch into larvae during the next few days. The food source, photoperiod, temperature and relative humidity affect the development of the mosquito at various stages of its life cycle^65^. During the developmental stages, the food consisted of high carbohydrate source with a mixture of yeast extract: dog biscuit: 10% sucrose solution (1:3:1), every day twice at relative interval. A photoperiod of 14 h of daylight and 10 h of darkness was maintained in the laboratory during the course of entire experiment. The optimum temperature was maintained at 27 ± 2 °C with 70 ± 9% relative atmospheric humid condition as per the methodology followed in the published literature^66^. Once the eggs hatched in water, mosquito larvae were transferred into a larger glass beaker with 1000 ml distilled water and food supplementation for rearing. The late 3rd or early 4th instar stage of mosquito larvae were used for in vitro AChE inhibition assay as well as for in vivo larvicidal activity analysis.

### In vitro acetylcholinesterase inhibition assay

For the AChE inhibition biochemical assay, enzyme was prepared by homogenizing *Cx. pipiens* larvae using 0.05 M phosphate buffer, pH 8.0 at 4 °C by using a homogenizer. The homogenate was centrifuged at 10,000 rpm for 15 min at 4 °C. The amount of total protein was estimated^67^ from the crude enzyme and the collected supernatant was then used as an enzyme source for the assay.

AChE estimation assay was carried out through the optimised protocol by Ellman published in the year 1961 with some minor modifications^68^. The chemical components include (i) phosphate buffer (0.1 M, pH 8.0), (ii) the substrate acetylthiocholine iodide (0.1 M), (Sisco Research Laboratories Pvt. Ltd. (SRL), India) (iii) Ellman's reagent (5,5'-dithiobis-(2-nitrobenzoic acid) or DTNB (0.01 M) (Sisco Research Laboratories Pvt. Ltd. (SRL), India) dissolved in phosphate buffer (0.1 M, pH 8.0) and (iv) the crude enzyme. The typical reaction mixture was prepared of 300.00 µl of buffer, 2.00 µl of substrate, 10.00 µl DTNB and 5.00 µl of enzyme. The blank for such a run consists of buffer, substrate and DTNB solution, while the control included every other reaction component except the substrate.

Furthermore, inhibitors were introduced in the reaction mixture for AChE inhibition assay. Pyridostigmine bromide was used as reference positive control while analytical grade malathion of brand name pestanal (Sigma-Aldrich Co, India) and curcumin (Sigma-Aldrich Co., India) were the test inhibitors for this study and their AChE inhibition activity was determined using their various concentration ranging from 10 to 250 µM. Change in absorbance was monitored at 412 nm for 13 min in a microplate reader (Synergy H1 Hybrid Multi-Mode Microplate Reader, USA) and the assay was replicated thrice. Percentage AChE inhibition was calculated according to the following formula:

2 $$ \% \;AChE\;Inhibition = \left[ {1 - \left( {sample\;reaction\;rate/blank\;reaction\;rate} \right)} \right] \times 100 $$

The rate of reaction, V (µmoles/L/min) for each inhibitor concentration was determined at various substrate concentrations [S] using the Michaelis–Menten plot and the nature of inhibition was speculated from the double reciprocal Lineweaver-Burke plot^69^. The IC_50_ value was calculated using the log-Probit analysis.

### In vivo larval mortality assessment and AChE inhibition

Larvicidal bioassay was performed using late 3rd and early 4th instar larvae of *Cx. pipiens*, following the WHO prescribed guidelines for laboratory testing of larvicides^70^. 0.05 M stock solution of curcumin and malathion were prepared, using which several working concentrations (50 to 250 µM) for both test compound was made. Bioassay was performed in a six-well plate (with lid 127.8 × 85.5 × 23.2 mm) and ten *Cx. pipiens* larvae were introduced into each well. Each plate had one well with 1 mL of acetone added with distilled water which served as control. All bioassays were carried out in a laboratory setup maintained at a temperature of 27 ± 2 °C with 70 ± 9% relative humidity. Mortality of the tested larvae was recorded after 24 h of exposure. Larval mortality was recorded, and percentage mortality was calculated using the formula:

3 $$ \% \;Larvae\;mortality = Number\;of\;dead\;larvae \times 100/Total\;number\;of\;larvae $$

Each treatment was replicated thrice and the average larval mortality data was subjected to Probit analysis^71^ for calculating LC_50_. Simultaneously, in vivo AChE inhibition assay was performed by exposing the larvae for 30 min with the same working concentrations (ranging from 50 to 250 µM) of curcumin and malathion as inhibitors. After the exposure time, larvae were recovered and homogenized for subsequent AChE enzyme assay.

## Data Availability

All the relevant data is contained within the manuscript. Additional raw data will be available upon request.

## References

[1] WHO (2018). Global Vector Control Response 2017–2030.

[2] Rao P, Goswami D, Rawal R (2021). Cry toxins of Bacillus thuringiensis: A glimpse into the Pandora’s box for the strategic control of vector borne diseases. Environ. Sustain..

[3] Ewing DA, Purse BV, Cobbold CA, Schäfer SM, White SM (2019). Uncovering mechanisms behind mosquito seasonality by integrating mathematical models and daily empirical population data: Culex pipiens in the UK. Parasites Vectors.

[4] Arora S, Balotra S, Pandey G, Kumar A (2017). Binary combinations of organophosphorus and synthetic pyrethroids are more potent acetylcholinesterase inhibitors than organophosphorus and carbamate mixtures: An in vitro assessment. Toxicol. Lett..

[5] Yu SJ (2011). The toxicology and biochemistry of insecticides. Toxicol. Biochem. Insectic..

[6] Hirata K (2016). Studies on the mode of action of neurotoxic insecticides. J. Pestic. Sci..

[7] Gajger IT, Dar SA (2021). Plant allelochemicals as sources of insecticides. Insects.

[8] Shaalan EAS, Canyon D, Younes MWF, Abdel-Wahab H, Mansour AH (2005). A review of botanical phytochemicals with mosquitocidal potential. Environ. Int..

[9] Soares Rodrigues GC (2021). Computer-assisted discovery of compounds with insecticidal activity against *Musca domestica* and *Mythimna separata*. Food Chem. Toxicol..

[10] Parmar P (2021). Meticulous assessment of natural compounds from NPASS database for identifying analogue of GRL0617, the only known inhibitor for SARS-CoV2 papain-like protease (PLpro) using rigorous computational workflow. Mol. Divers..

[11] Rao P, Shukla A, Parmar P, Goswami D (2020). Proposing a fungal metabolite-Flaviolin as a potential inhibitor of 3CLpro of novel coronavirus SARS-CoV2 using docking and molecular dynamics. J. Mol. Dyn..

[12] Rao P (2020). Reckoning a fungal metabolite, Pyranonigrin A as a potential Main protease (Mpro) inhibitor of novel SARS-CoV-2 virus identified using docking and molecular dynamics simulation. Biophys. Chem..

[13] Rao P (2021). Identifying structural–functional analogue of GRL0617, the only well-established inhibitor for papain-like protease (PLpro) of SARS-CoV2 from the pool of fungal metabolites using docking and molecular dynamics simulation. Mol. Divers..

[14] Mohanraj K (2018). IMPPAT: A curated database of Indian Medicinal Plants, Phytochemistry and Therapeutics. Sci. Rep..

[15] Madeira F (2019). The EMBL-EBI search and sequence analysis tools APIs in 2019. Nucleic Acid Res..

[16] Khan HAA, Shad SA, Akram W (2013). Resistance to new chemical insecticides in the house fly, *Musca domestica* L., from dairies in Punjab, Pakistan. Parasitol. Res..

[17] McCarroll L (2000). Insecticides and mosquito-borne disease: Insecticide resistance in mosquitoes can also interfere with developing parasites. Nature.

[18] Silver KS (2014). Voltage-gated sodium channels as insecticide targets. Adv. Insect Physiol..

[19] Ramos RS (2019). Potential inhibitors of the enzyme acetylcholinesterase and juvenile hormone with insecticidal activity: Study of the binding mode via docking and molecular dynamics simulations. J. Biomol. Struct. Dyn..

[20] Casida JE, Durkin KA (2015). Novel GABA receptor pesticide targets. Pestic. Biochem. Physiol..

[21] Francis S (2017). Insecticide resistance to permethrin and malathion and associated mechanisms in *Aedes aegypti* mosquitoes from St. Andrew Jamaica. PLoS ONE.

[22] Yamagishi Y, Iwase H, Ogra Y (2021). Effects of human serum albumin on post-mortem changes of malathion. Sci. Rep..

[23] Singh KD (2017). Biochemical efficacy, molecular docking and inhibitory effect of 2, 3-dimethylmaleic anhydride on insect acetylcholinesterase. Sci. Rep..

[24] Yerdelen KO, Tosun E (2015). Synthesis, docking and biological evaluation of oxamide and fumaramide analogs as potential AChE and BuChE inhibitors. Med. Chem. Res..

[25] Genheden S, Ryde U (2015). The MM/PBSA and MM/GBSA methods to estimate ligand-binding affinities. Expert Opin. Drug Discov..

[26] Li J (2011). The VSGB 2.0 model: A next generation energy model for high resolution protein structure modeling. Proteins Struct. Funct. Bioinform..

[27] Patel CN (2021). Pinpointing the potential hits for hindering interaction of SARS-CoV-2 S-protein with ACE2 from the pool of antiviral phytochemicals utilizing molecular docking and molecular dynamics (MD) simulations. J. Mol. Graph. Model..

[28] Goswami D, Patel CN, Goswami D, Sivakumar PK, Pandya HA (2021). Repurposing of anticancer phytochemicals for identifying potential fusion inhibitor for SARS-CoV-2 using molecular docking and molecular dynamics (MD) simulations. J. Biomol. Struct. Dyn..

[29] Keretsu S, Bhujbal SP, Cho SJ (2020). Rational approach toward COVID-19 main protease inhibitors via molecular docking, molecular dynamics simulation and free energy calculation. Sci. Rep..

[30] Yoshino R, Yasuo N, Sekijima M (2020). Identification of key interactions between SARS-CoV-2 main protease and inhibitor drug candidates. Sci. Rep..

[31] Rao P (2020). Proposing a fungal metabolite-Flaviolin as a potential inhibitor of 3CLpro of novel coronavirus SARS-CoV-2 identified using Docking and Molecular Dynamics. J. Biomol. Struct. Dyn..

[32] Komatsu TS (2020). Drug binding dynamics of the dimeric SARS-CoV-2 main protease, determined by molecular dynamics simulation. Sci. Rep..

[33] Murugan NA, Kumar S, Jeyakanthan J, Srivastava V (2020). Searching for target-specific and multi-targeting organics for Covid-19 in the Drugbank database with a double scoring approach. Sci. Rep..

[34] Bowers, K. J. *et al.* Scalable algorithms for molecular dynamics simulations on commodity clusters. In *Proceedings of the 2006 ACM/IEEE Conference on Supercomputing, SC’06* 84 (ACM Press, 2006). 10.1145/1188455.1188544.

[35] Chandra Roy G, Chakraborty K, Nandy P, Moitra MN (2014). Pros and cons of curcumin as bioactive phyto-compound for effective management of insect pests. Am. Sci. Res. J. Eng. Technol. Sci..

[36] Sagnou M (2012). Evaluation of naturally occurring curcuminoids and related compounds against mosquito larvae. Acta Trop..

[37] Abbasi MA (2012). Curcumin and its derivatives: Moderate inhibitors of acetylcholinesterase, butyrylcholinesterase and trypsin. Sci. Iran..

[38] Salehi B (2019). The therapeutic potential of curcumin: A review of clinical trials. Eur. J. Med. Chem..

[39] Hamaguchi T, Ono K, Yamada M (2010). Curcumin and Alzheimer’s disease. CNS Neurosci. Ther..

[40] Simeonova R (2021). A novel galantamine-curcumin hybrid as a potential multi-target agent against neurodegenerative disorders. Molecules.

[41] Verdín-Betancourt FA (2019). In vitro inhibition of human red blood cell acetylcholinesterase (AChE) by temephos-oxidized products. Sci. Rep..

[42] Shen L, Liu C-C, An C-Y, Ji H-F (2016). How does curcumin work with poor bioavailability? Clues from experimental and theoretical studies. Sci. Rep..

[43] Renuga Parameswari A, Rajalakshmi G, Kumaradhas P (2015). A combined molecular docking and charge density analysis is a new approach for medicinal research to understand drug-receptor interaction: Curcumin-AChE model. Chemico-Biol. Interact..

[44] Saravanan K, Kalaiarasi C, Kumaradhas P (2017). Understanding the conformational flexibility and electrostatic properties of curcumin in the active site of rhAChE via molecular docking, molecular dynamics, and charge density analysis. J. Biomol. Struct. Dyn..

[45] Bateman A (2019). UniProt: A worldwide hub of protein knowledge. Nucleic Acids Res..

[46] Waterhouse A (2018). SWISS-MODEL: Homology modelling of protein structures and complexes. Nucleic Acids Res..

[47] Burley SK (2021). RCSB Protein Data Bank: Powerful new tools for exploring 3D structures of biological macromolecules for basic and applied research and education in fundamental biology, biomedicine, biotechnology, bioengineering and energy sciences. Nucleic Acids Res..

[48] Berman HM (2000). The Protein Data Bank. Nucleic Acids Res..

[49] Studer G (2020). QMEANDisCo—distance constraints applied on model quality estimation. Bioinformatics.

[50] Chen VB (2010). MolProbity: All-atom structure validation for macromolecular crystallography. Acta Crystallogr. Sect. D Biol. Crystallogr..

[51] Tian W, Chen C, Lei X, Zhao J, Liang J (2018). CASTp 3.0: Computed atlas of surface topography of proteins. Nucleic Acids Res..

[52] Madhavi Sastry G, Adzhigirey M, Day T, Annabhimoju R, Sherman W (2013). Protein and ligand preparation: Parameters, protocols, and influence on virtual screening enrichments. J. Comput. Aided. Mol. Des..

[53] Jorgensen WL, Tirado-Rives J (1988). The OPLS potential functions for proteins. Energy minimizations for crystals of cyclic peptides and crambin. J. Am. Chem. Soc..

[54] Jorgensen WL, Maxwell DS, Tirado-Rives J (1996). Development and testing of the OPLS all-atom force field on conformational energetics and properties of organic liquids. J. Am. Chem. Soc..

[55] Shivakumar D (2010). Prediction of absolute solvation free energies using molecular dynamics free energy perturbation and the OPLS force field. ACS Publ..

[56] Mohanraj K (2017). IMPPAT: A curated database of Indian Medicinal plants phytochemistry and therapeutics. bioRxiv.

[57] Kim S (2021). PubChem in 2021: New data content and improved web interfaces. Nucleic Acids Res..

[58] Shelley JC (2007). Epik: A software program for pKa prediction and protonation state generation for drug-like molecules. J. Comput. Aided. Mol. Des..

[59] Greenwood JR, Calkins D, Sullivan AP, Shelley JC (2010). Towards the comprehensive, rapid, and accurate prediction of the favorable tautomeric states of drug-like molecules in aqueous solution. J. Comput. Aided Mol. Des..

[60] Massova I, Kollman PA (2000). Combined molecular mechanical and continuum solvent approach (MM- PBSA/GBSA) to predict ligand binding. Perspect. Drug Discov. Des..

[61] Wang W, Donini O, Reyes CM, Kollman PA (2001). Biomolecular simulations: Recent developments in force fields, simulations of enzyme catalysis, protein-ligand, protein-protein, and protein-nucleic acid noncovalent interactions. Annu. Rev. Biophys. Biomol. Struct..

[62] Jacobson MP, Friesner RA, Xiang Z, Honig B (2002). On the role of the crystal environment in determining protein side-chain conformations. J. Mol. Biol..

[63] Jacobson MP (2004). A hierarchical approach to all-atom protein loop prediction. Proteins Struct. Funct. Genet..

[64] Darden T, York D, Pedersen L (1993). Particle mesh Ewald: An N·log(N) method for Ewald sums in large systems. J. Chem. Phys..

[65] Kauffman E (2017). Rearing of Culex spp. and Aedes spp. mosquitoes. Bio-Protoc..

[66] Das S, Garver L, Dimopoulos G (2007). Protocol for mosquito rearing (*A. gambiae*). J. Vis. Exp..

[67] Bradford M (1976). A rapid and sensitive method for the quantitation of microgram quantities of protein utilizing the principle of protein-dye binding. Anal. Biochem..

[68] Ellman GL, Courtney KD, Andres V, Featherstone RM (1961). A new and rapid colorimetric determination of acetylcholinesterase activity. Biochem. Pharmacol..

[69] Lineweaver H, Burk D (1934). The determination of enzyme dissociation constants. J. Am. Chem. Soc..

[70] WHO (2005). Guidelines for Laboratory and Field Testing of Mosquito larvicides.

[71] Brownlee KA, Finney DJ, Tattersfield F (1952). Probit analysis: A statistical treatment of the sigmoid response curve. J. Am. Stat. Assoc..

